# Supplementary material for: Molecular and Functional Characteristics of Airway Epithelium under Chronic Hypoxia
Source: Int J Mol Sci. 2023 Mar 30;24(7):6475. doi: 10.3390/ijms24076475 (PMC10095024; doi:10.3390/ijms24076475)
Supplement: Supplementary file 1 [file ijms-24-06475-s001.zip › Supplementary materials_Wong et al_Submission.pdf]

Supplementary materials

Title: Molecular and functional characteristics of airway epithelium under chronic hypoxia.

Sharon L. Wong, Egi Kardia, Abhishek Vijayan, Bala Umashankar, Elvis Pandzic, Ling Zhong, Adam Jaffe and Shafagh A. Waters

Table S1. List of differentially expressed proteins (DEPs) for hNECs conditional reprogramming cultures. (Excel file)

Table S2. List of differentially expressed proteins (DEPs) for hNECs air-liquid interface cultures. (Excel file)

Table S3. IPA pathway analysis of differentially expressed proteins for hNECs conditional reprogramming cultures. (Excel file)

Table S4. IPA pathway analysis of differentially expressed proteins for hNECs air-liquid interface cultures. (Excel file)

Table S5. Details of study participants

**Participant ID**	***CFTR* genotype**	**Age (yr)**	**Sex**
WT1	WT/WT	2.5	M
WT2	WT/WT	1.4	Unknown
WT3	WT/WT	13.3	M
CF1	F508del/F508del	11.2	M
CF2	F508del/F508del	5.0	M
CF3	F508del/F508del	13.8	F
CF4	F508del/F508del	6.4	F
CF5	F508del/F508del	14.2	M
CF6	F508del/F508del	3.7	M
CF7	F508del/F508del	4.8	F
CF8	F508del/F508del	4.5	F

Table S6. Antibodies for immunofluorescence staining

Antibody	Dilution	Supplier	Catalogue number
Rabbit monoclonal anti-E-cadherin (24E10)	1:100	Cell Signalling	3195
Mouse monoclonal anti-MUC5AC (45M1)	1:250	Life Technologies	MA5-12178
Rabbit monoclonal anti-Acetyl-α-Tubulin (Lys40) (D20G3) XP, Alexa Fluor 647 conjugate	1:50	Cell Signalling	81502
Goat polyclonal anti-mouse IgG antibody, Alexa Fluor 555	1:500	Life Technologies	A-21424
Goat polyclonal anti-rabbit IgG antibody, Alexa Fluor 488	1:500	Life Technologies	A-11034

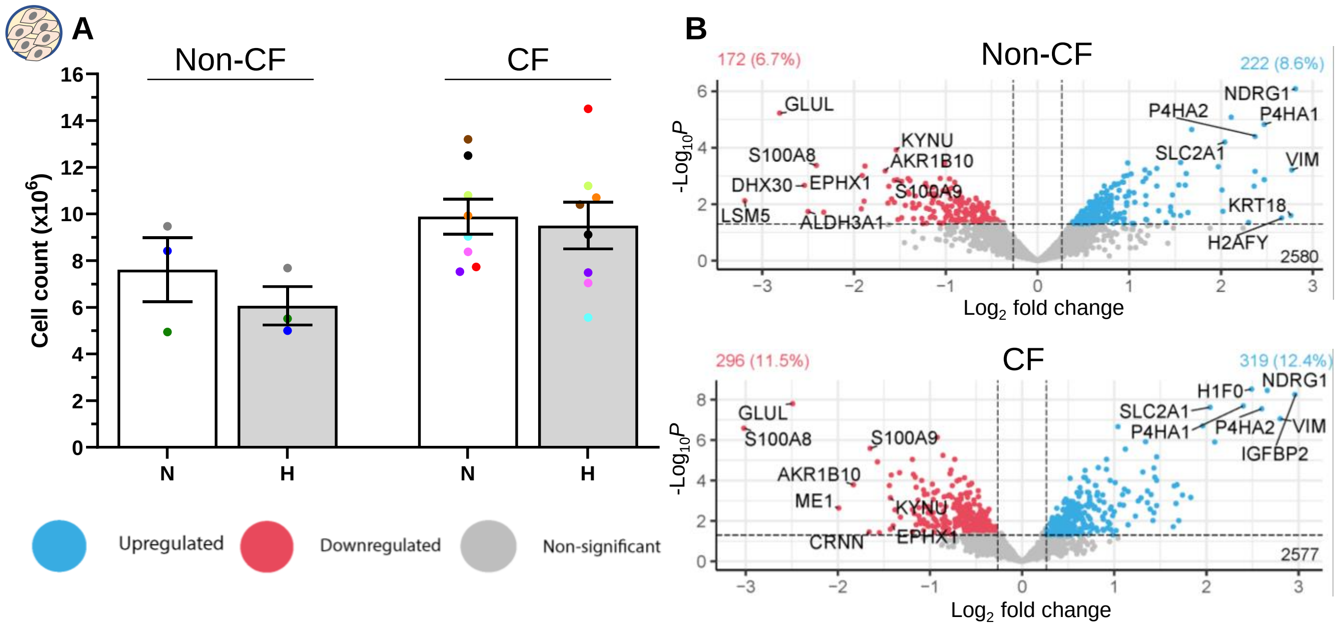

Figure S1. Effect of chronic hypoxia on the expansion of primary human nasal epithelial cells (hNECs). (A) Cell count of non-CF (n=3) and CF (n=8) hNECs following expansion under normoxic and chronic hypoxic conditions. Each coloured circle represents cultures from an individual participant. Data are presented as bar plots with mean ± standard error of the mean (SEM). One-way ANOVA was used to determine statistical significance. (B) Volcano plots of differentially expressed proteins (DEPs) in hypoxic cells compared to normoxic cells in non-CF (n=3) and CF (n=8). Dotted lines indicate significance cut-off (p-value ≤0.05, |fold change| ≥1.2). The count of significant upregulated proteins, significant downregulated proteins and total proteins are shown in top right, top left and bottom right respectively. The top 5 to 10 upregulated and downregulated proteins are labelled.

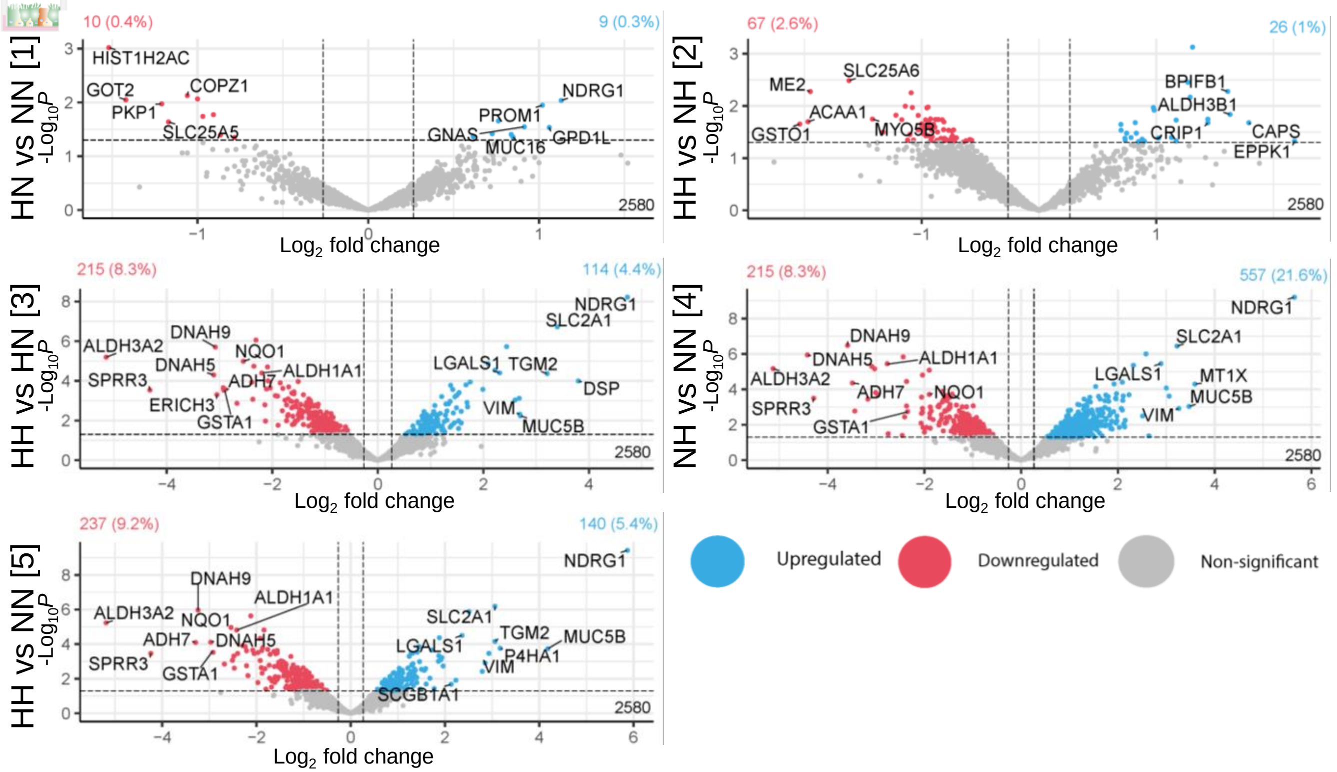

Figure S2. Effect of chronic hypoxia on the global proteome of differentiated human nasal epithelial cells (hNECs). Volcano plots of differentially expressed proteins (DEPs) in each oxygen condition in differentiated air-liquid interface (ALI) cultures from three non-CF participants. Dotted lines indicate significance cut-off (p-value ≤0.05, |fold change| ≥ 1.2). The count of significant upregulated proteins, significant downregulated proteins and total proteins are shown in top right, top left and bottom right respectively. The top 5 to 10 upregulated and downregulated proteins (determined based on logFC) are labelled. Comparisons are in pairs; [1] HN compared to NN; [2] HH compared to NH; [3] HH compared to HN; [4] NH compared to NN and [5] HH compared to NN.

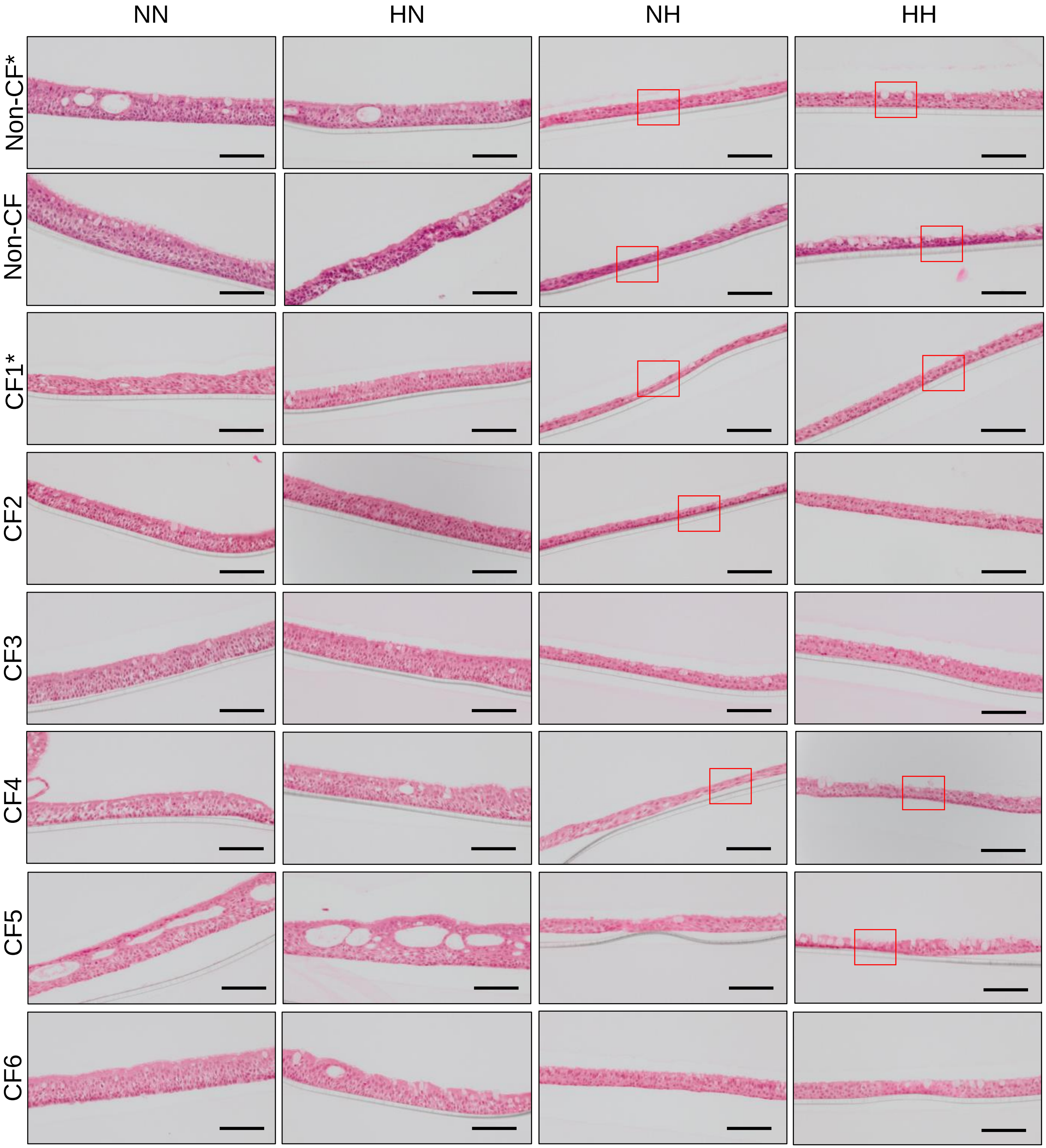

Figure S3. H&E stain of primary hNECS from two non-CF and six CF participants differentiated at ALI in normoxia (NN and HN) and chronic hypoxia (NH and HH) for 21-25 days. Red rectangle shows squamous cells or cells transitioning towards squamous morphology. *indicate donor images which are displayed in Figure 4 of the main manuscript. 40x/0.8 objective. Scale bars = 100 μm.

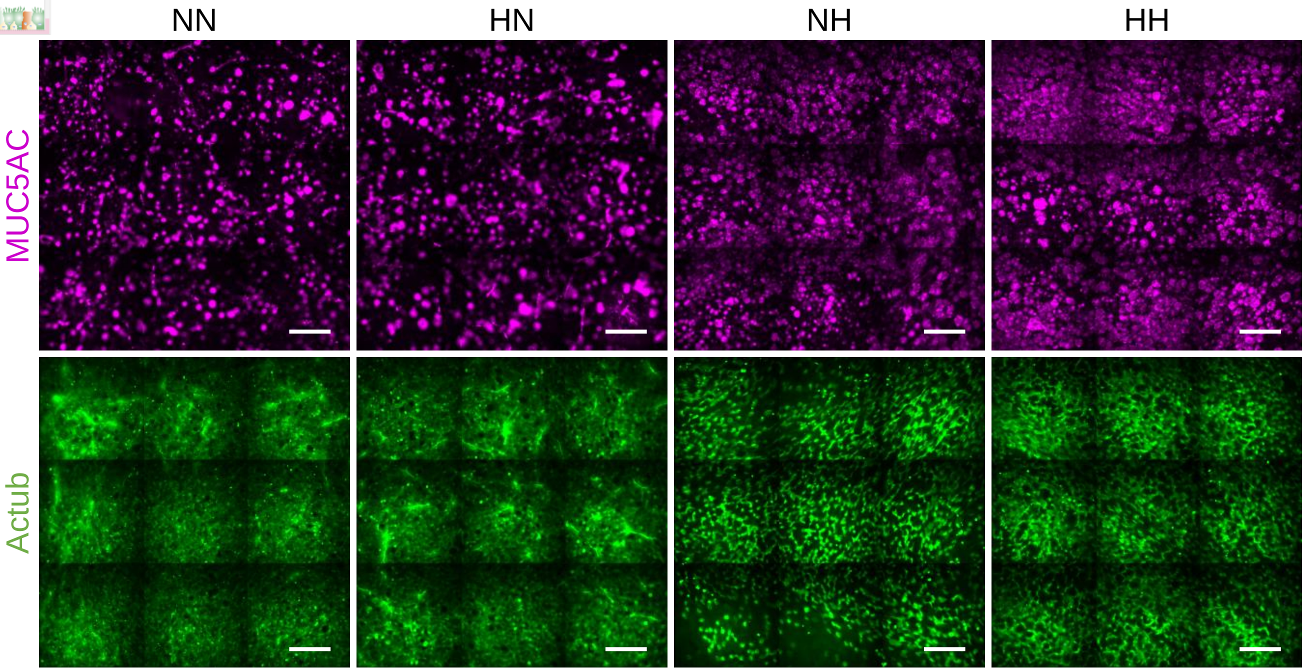

Figure S4. Tiled images of the whole membrane with immunofluorescence staining of MUC5AC (magenta) and acetylated tubulin (actub, green). 20x/0.8 objective. Scale bars = 100μm.
